# Effect of tungsten on the oxidation of alumina-forming austenitic stainless steel

**DOI:** 10.1186/s42649-019-0014-4

**Published:** 2019-11-14

**Authors:** Jun-Yun Kang, Heon-Young Ha, Sung-Dae Kim, Jun Young Park, Min-Ho Jang, Tae-Ho Lee

**Affiliations:** 1 Korea Institute of Materials Science, 797 Changwon-daero, Changwon, Gyeongnam 51508 Republic of Korea; 2 Present Address: Hyundai Steel, 1480, Bukbusaneop-ro, Dangjin-Si, Chungnam 31719 Republic of Korea

**Keywords:** Sainless steel, TEM, High temperature corrosion, Oxidation

## Abstract

As more W replaced Mo in alumina-forming austenitic stainless steels, weight gain by oxidation decreased after 336 h at 1053 K. Electron microscopy revealed slower growth of scale in the presence of more numerous second phases by W addition. The retardation of oxidation was attributed to the necessary partitioning of W in front of the metal-oxide interface. The W-rich second phases interacted with growing oxides and finally transformed to fine particles of metallic W alloy within the scale.

## Introduction

Many variants of austenitic stainless steels have been common materials in building various components which required long-term structural stability upon exposure to high temperature. The key properties for these applications are creep and oxidation-resistance. And enormous efforts have been devoted to their improvement in order to cope with the increasing service temperatures (Mayer and Masuyama 2008; Igarashi 2008).

The oxidation-resistance of conventional austenitic stainless steels is obtained by the formation of chromia (Cr_2_O_3_) layer as the protective surface scale. On the other hand, a considerable number of attempts have been made to substitute it with alumina (Al_2_O_3_) as a way to improve oxidation-resistance (Ramakrishnan et al. 1988; Yamamoto et al. 2007a; Yamamoto et al. 2007b; Brady et al. 2008). The latter has higher thermodynamic stability in oxygen, much slower growth rate and does not exhibit volatility in wet air (Yamamoto et al. 2007a; Yamamoto et al. 2007b; Brady et al. 2008; Opila 2004). Thus, the recent achievement in the development of alumina-forming austenitic stainless steels (AFA) (Yamamoto et al. 2007a; Yamamoto et al. 2007b; Brady et al. 2008) has attracted a number of subsequent investigates to assess their feasibility in various applications (Pint et al. 2013; Brady et al. 2013; He et al. 2014; Ejenstam and Szakálos 2015; Put et al. 2015) and to improve their properties (Brady et al. 2009; Yamamoto et al. 2009a; Brady et al. 2011a; Yamamoto et al. 2011; Yamamoto et al. 2013) as well as cost efficiency (Yamamoto et al. 2009b; Brady et al. 2014).

Alloying with Mo and W have been frequently used to enhance the performance of various steels for high-temperature applications (Mayer and Masuyama 2008; Igarashi 2008; Liu and Fujita 1988; Tsuchida et al. 1995; Miyata and Sawaragi 2001; Cui et al. 2011; Jang et al. 2015; Hosoi et al. 1996; Yun et al. 2011; Yun et al. 2012), while their effects on creep-resistance have been more focused (Igarashi 2008; Liu and Fujita 1988; Tsuchida et al. 1995; Miyata and Sawaragi 2001; Cui et al. 2011; Jang et al. 2015). They are transition metals belonging to the same group VI in periodic table, have approximately identical atomic radius (2.17 and 2.18 Å respectively) (Periodic table from the Royal Society of Chemistry n.d.), thus are usually regarded to play parallel roles in Fe-base alloys. Based on this perception, the following Mo equivalent which was the normalized weight fraction by approximate ratio of their atomic mass has been widely used by steel metallurgists (Liu and Fujita 1988; Tsuchida et al. 1995; Miyata and Sawaragi 2001; Cui et al. 2011; Jang et al. 2015).

1 $$ {\mathrm{Mo}}_{\mathrm{eq}}=\left[\mathrm{wt}.\%\mathrm{of}\ \mathrm{Mo}\right]+0.5\left[\mathrm{wt}.\%\mathrm{of}\ \mathrm{W}\right] $$

With regard to the comparison of Mo and W effect, several works indicated superior effectiveness of W on creep-resistance (Igarashi 2008; Liu and Fujita 1988; Tsuchida et al. 1995; Miyata and Sawaragi 2001; Cui et al. 2011; Jang et al. 2015). In fixed Mo_eq_, increased W (consequently decreased Mo) generally increases creep rupture strength or time to rupture via modification of precipitates (Igarashi 2008; Tsuchida et al. 1995; Miyata and Sawaragi 2001; Cui et al. 2011; Jang et al. 2015). On the other hand, a more limited number of works (Hosoi et al. 1996; Yun et al. 2011; Yun et al. 2012) were found about their effect on oxidation-resistance. Although both elements were positively effective up to certain amounts of addition (Hosoi et al. 1996; Yun et al. 2011; Yun et al. 2012), the comparison of their effectiveness was not presented.

In this work, oxidation behaviors of AFAs with varying Mo and W contents were analyzed and superior effectiveness of W on oxidation-resistance was observed. Therefore, detailed microscopic works on the characteristic features of oxide layers and interfaces are presented to address the effect of W in comparison with Mo, which should contribute to further optimization and evolution of AFAs.

## Material and methods

One of the original AFAs from (Yamamoto et al. 2007a) and its variants with varying Mo and W contents were prepared. The target and the measured compositions are listed in Table 1. Following the target composition, Mo_eq_ was controlled to be close to 2.5. The designations of the alloys, i.e. W0-W5 describe their W contents which substituted for equivalent Mo contents.

**Table 1:** Chemical compositions of the alloys (in wt.%)

Alloy	C	Si	Mn	Al	Cr	Ni	Mo	W	Mo_eq_	Nb	
Target	0.08	0.15	2.00	2.50	14.00	20.00	Mo_eq_ = 2.50			0.85	B: 0.01 P: 0.01 Fe: Bal.
W0	0.08	0.16	2.02	2.45	14.28	20.74	2.46	0.01	2.47	0.82	
W1	0.08	0.16	1.99	2.45	14.25	20.48	2.06	0.89	2.50	0.81	
W2	0.08	0.15	1.95	2.53	14.15	20.21	1.69	1.77	2.57	0.85	
W3	0.08	0.15	1.94	2.60	14.07	19.95	1.02	3.07	2.56	0.86	
W4	0.08	0.15	1.92	2.57	14.03	19.84	0.88	3.65	2.71	0.85	
W5	0.08	0.15	1.93	2.57	14.01	19.66	0.41	4.63	2.73	0.82	

Cast ingots by vacuum induction melting were reheated to 1523 K, held for 2 h, hot rolled between 1373 and 1173 K by thickness reduction of 50% and quenched in water. Then, the hot rolled plates were cold rolled by thickness reduction of 60%, annealed at 1523 K for an hour and quenched. Finely polished surfaces of the annealed specimens were exposed to hot atmosphere kept at 1053 K for up to 336 h. And weight gains by oxidation were measured.

The microstructures around surface scales were analyzed on two representative specimens, i.e. W0 and W5 which had the minimum and the maximum W contents respectively. A field emission scanning electron microscope (SEM, JSM-7001F by JEOL) and a field emission transmission electron microscope (TEM, JEM-2100F by JEOL) were used. Electron-transparent specimens for TEM were fabricated by a focused ion beam system (FIB, JIB-4601F by JEOL).

## Results

**Fig. 1:**
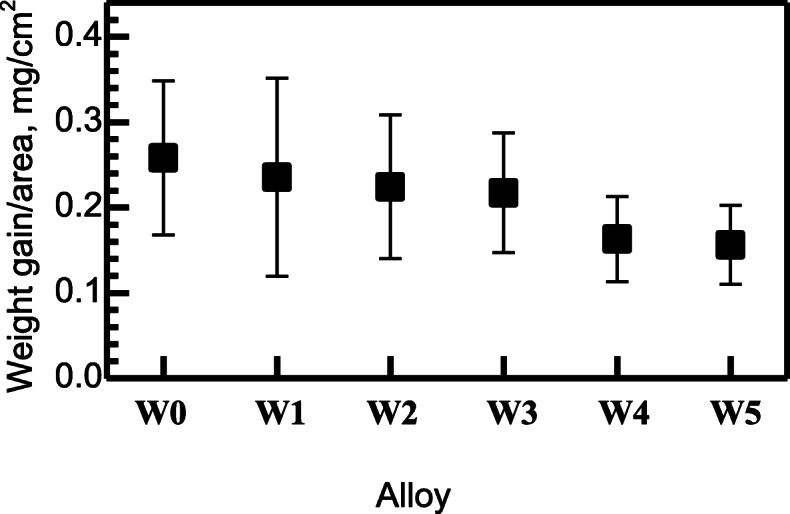
Weight gain of the alloys after 336 h of oxidation at 1053 K (error bars represent standard deviations)

Weight gains according to the alloy compositions after 336 h of oxidation are presented in Fig. 1. As W content increased, i.e. substituted for more equivalent content of Mo, weight gains continued to decrease. Thus, at least for the given conditions and durations of oxidation, it could be said that W enhanced oxidation-resistance more effectively than Mo.

**Fig. 2:**
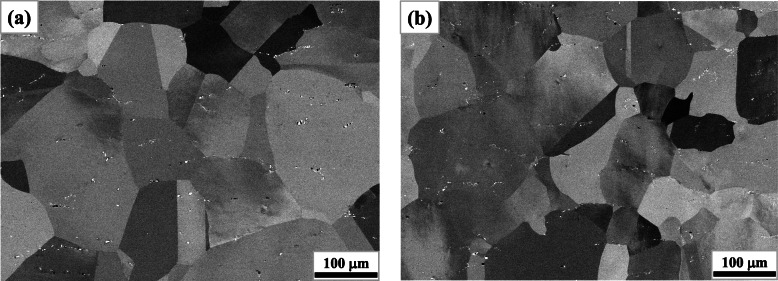
Initial microstructures prior to oxidation: **a** W0, **b** W5

The initial microstructures prior to oxidation exhibited negligible differences according to the alloy compositions, which is well represented in Fig. 2. It presents the initial microstructures of W0 and W5, both of which exhibited typical microstructures of AFAs (Pint et al. 2013; Yamamoto et al. 2011; Jang et al. 2015) by annealing and homogenization at high temperatures (1523 K in the present work). They had austenitic matrix of comparable grain sizes (approximately 100 μm in average) with undissolved primary Nb(C,N) particles (the bright spots in Fig. 2) of similar distribution.

**Fig. 3:**
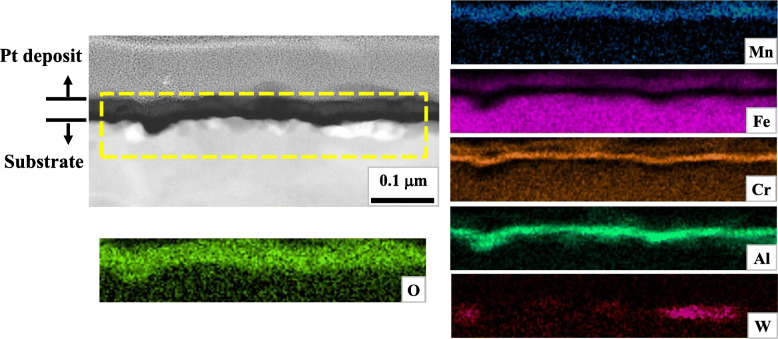
Microstructure around the scale of W5 after 30 min of oxidation (STEI-DF and elemental maps by EDS within the broken box)

Figure 3 presents a scanning transmission electron image in dark field mode (STEI-DF) and elemental distribution maps by energy dispersive spectroscopy (EDS) around the scale of W5 after a short duration (30 min) of oxidation. They represent the general constitution of the oxide scale. It could be known that the scale consisted of multiple layers of different compositions, which led to the varying brightness of the scale in the micrographs by compositional contrast. The outermost layer was (Fe, Mn, Cr)-rich oxide beneath which more Cr-enriched layer existed. The darker inner layer adjacent to the metallic (austenitic) substrate was Al-rich oxide, i.e. alumina. This multi-layered structure of scale well accords with some previous observations on the scale of AFAs (He et al. 2014; Brady et al. 2011a; Kang et al. 2015; Brady et al. 2011b; Rother et al. 2012). Beneath the scale of W5, there was a characteristic region in which dark and bright volumes alternated. And the W map in Fig. 3 shows that the bright volume is rich in W and indicates the existence of W-rich second phases. In Table 2, the average compositions of the two characteristic features (dark and bright) are presented with those of the alumina layer and the metallic matrix (100 μm below the interface between the substrate and the scale). All of them were measured by EDS, thus it should be noted that a considerable deviation from the actual composition was inevitable due to limited spatial resolution. Albeit this, it was apparent that the dark volume was much richer in O and Al and more deficient in Cr than the matrix. On the other hand, the dark volume exhibited much lower O and Al content than the alumina layer whilst Fe and Ni contents were close to those of the matrix.

**Table 2:** Average compositions of the characteristic features in Fig. 3 measured by EDS (at. %)

Feature	O	Al	Si	Cr	Mn	Fe	Ni	Nb	Mo	W
Alumina	48.62	41.88	1.16	1.03	0.00	4.26	2.00	0.66	0.18	0.21
Matrix	0.91	3.09	0.00	15.55	0.80	58.05	18.94	0.84	0.41	1.41
Dark	17.41	11.89	0.73	6.98	0.00	43.79	16.20	0.44	0.81	1.76
Bright	4.99	1.43	1.40	8.87	0.00	55.74	17.61	0.17	1.92	7.87

**Fig. 4:**
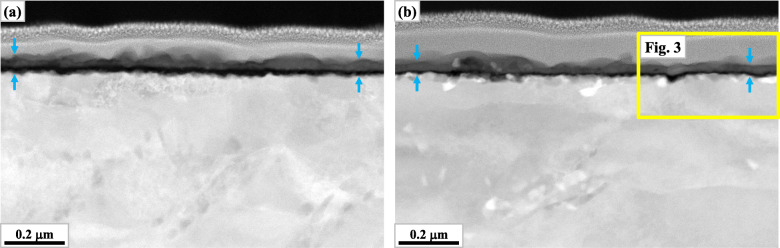
Microstructures around the scales after 30 min of oxidation (STEI-DF): **a** W0, **b** W5 (the scale region is indicated by the arrows and the area within the solid box corresponds to a part of Fig. 3)

**Fig. 5:**
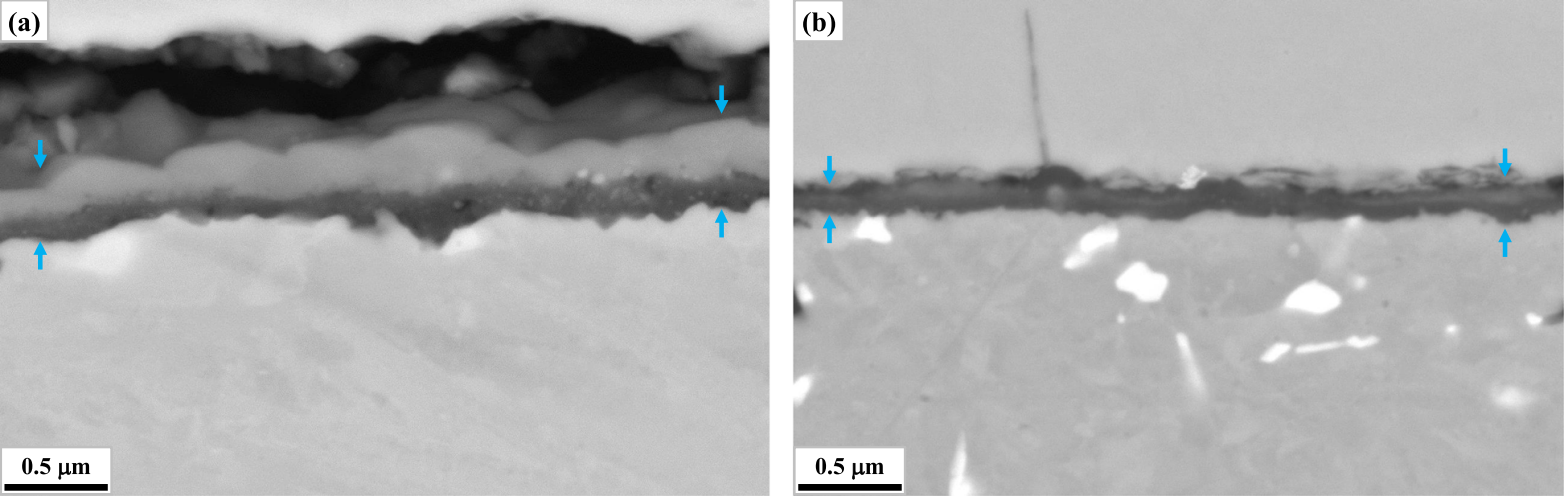
Microstructures around the scales after 336 h of oxidation (SEM-BEI): **a** W0, **b** W5

Figures 4 and 5 represent the microstructures around the scale of W0 and W5 after 30 min and 336 h of oxidation respectively. In Fig. 4b, a part of Fig. 3 is included. And in Fig. 5, backscattered electron images from the SEM (SEM-BEI) are presented instead of STEI-DFs in the consideration of substantial scale growth, while they are similar in the characteristics of image contrast with STEI-DFs. As can be deduced from Fig. 1, the increased W content retarded the growth of scales from the very early stages of the oxidation test. As shown in Fig. 4, alumina layer (the continuous darkest layer) was evidently thinner in W5 after 30 min of oxidation. And overall thickness of scale was thinner in W5 after 336 h, as presented in Fig. 5. It should be also noted that there were much more bright second phases in W5 from the early stage. They were absent in W0 after 30 min and only a few were found along the interface between the metallic substrate and the oxide scale as shown in Figs. 4a and 5a respectively. By contrast, a considerable number of them were found along the metal-oxide interface in W5 after 30 min, although they were hardly found in the matrix as shown in Fig. 4b. After prolonged duration, the matrix of W5 was also populated with them as shown in Fig. 5b.

**Fig. 6:**
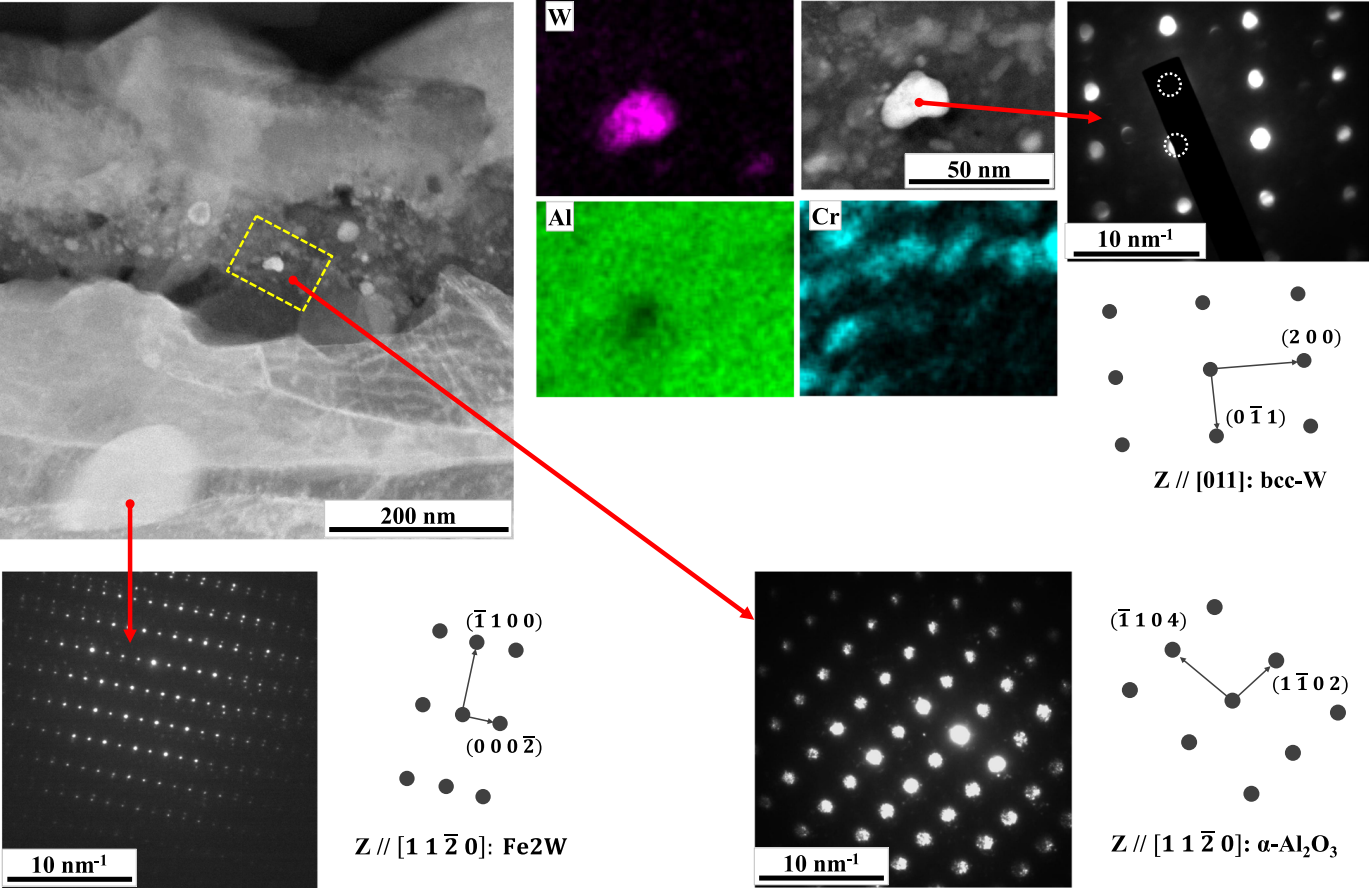
Analyses on the oxides and the second phases near the metal-oxide interface of W5 after 336 h of oxidation

Figure 6 provides more detailed information on the microstructure around the scale of W5 after 336 h. The local chemistry by EDS and the symmetry and spacing of the spots in selected area diffraction patterns (SADP) by nano-beam diffraction method presented the identification of the oxides and the second phases. The innermost Al-rich layer was definitely identified as alumina (α-Al_2_O_3_) while a coarse bright second phase beneath the metal-oxide interface was identified as Laves-phase. Between the outermost layer which was known as (Fe, Cr, Mn)-rich spinel (Brady et al. 2011a; Kang et al. 2015) and the innermost alumina layer, there was an intermediate layer in which many bright granules were embedded in the dark alumina layer. Most of the granules were richer in Cr than the surrounding alumina. Some distinctly brighter particles were frequently found inside the scale of W5 as represented within the broken box in the micrograph of Fig. 6. The identification of them would be the most interesting finding in Fig. 6. The brightest second phase was rich in W as shown in the W map. In addition, the average composition of these types of particles by EDS is also presented in Table 3. Although it exhibited high O and Al contents due to surrounding alumina layer, the ratio of W to other metallic contents were distinctly higher than in the other bright W-rich second phases beneath the metal-oxide interface. Its SADP showed the typical symmetry of body centered cubic (bcc) lattice with lattice parameter of about 3 Å, which very well accords with metallic W-based alloys of bcc lattices (Grum-Grzhimailo and Prokof’ev 1961; Taylor and Doyle 1965).

**Table 3:** Average composition of the bright W-rich particles inside the scale of W5 by EDS (at. %)

O	Al	Si	Cr	Mn	Fe	Ni	Nb	Mo	W
48.84	24.78	0.00	3.18	0.39	2.63	0.32	1.19	2.83	15.84

## Discussion

From approximately the same initial microstructures which are represented in Fig. 2, the effect of initial microstructure could be disregarded in the following discussion on the influence of W. On the other hand, as shown in Figs. 4 and 5, the evolution of microstructure during oxidation at the given temperature (1053 K) varied with the amount of W which substitutes for Mo. Besides the decreasing thickness of scale, the most noticeable change by W alloying was the increased second phase both at the metal-oxide interface and within the matrix.

**Fig. 7:**
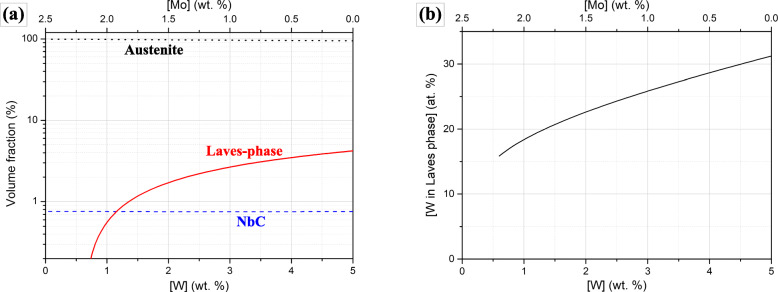
Thermodynamic equilibria in the substrate with the target composition (Mo_eq_ = 2.5) at 1053 K according to nominal W content: **a** phase equilibria, **b** equilibrium W content in Laves phase (note that the calculation with instable Laves-phase is indicated)

Figure 7 presents some calculations of thermodynamic equilibria in matrix at 1053 K. The target compositions in Table 1 with increasing W (i.e. decreasing Mo) contents were submitted to Thermo-Calc 3.0 software (Anderson et al. 2002) with TCFE7 database (Thermo-Calc Software TCFE7 Steels/Fe-alloys database version 7 n.d.). Figure 7a is the predicted volume fractions of equilibrium phases with W content, which shows the increasing Laves-phase with increasing W while the other second phase, i.e. NbC is hardly affected. In Fig. 7b, the equilibrium concentration of W in the increasing Laves-phase also increases with nominal W content. Ideally, the constitution of Laves phase should transform from Fe_2_Mo to Fe_2_W following Fig. 7b. Although both Mo and W were known as Laves-phase former (Igarashi 2008; Jang et al. 2015; Horita et al. 2008; Pavlů and Šob 2012; Lu et al. 2014), it could be known that W was more effective in the stabilization of Laves-phase when incorporated into it (Igarashi 2008; Jang et al. 2015; Lu et al. 2014). In Fig. 6, a W-rich Laves phase was identified below the metal-oxide interface of W5, which supported the thermodynamic predictions in Fig. 7.

**Fig. 8:**
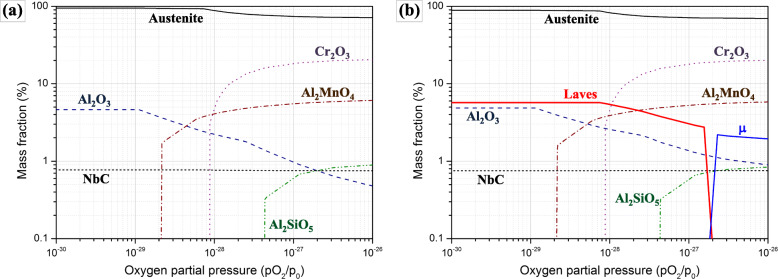
Phase equilibria at 1053 K according to oxygen partial pressure: **a** W0, **b** W5

Phase equilibria under oxidation could be also predicted with addition of database for various oxides. Phase fractions of W0 and W5 with oxygen partial pressure (pO_2_) were obtained by appending SSUB4 (Thermo-Calc Software SSUB4 Substances database version 4 n.d.) database to the above one (TCFE7). In the calculation, the measured compositions in Table 1 were used and the results under substantially low pO_2_ (10^− 30^ – 10^− 26^ atm, it should be noted that the equilibrium pO_2_ for alumina at 1273 K was only about 10^− 37^ atm for example (Khanna 2002).) are presented in Fig. 8 to approximate the reactions near substrate. In both alloys, alumina is preferred at low pO_2_ and other Cr, Mn or Si-containing oxides increases with increasing pO_2_, which exhibits good coherence with the observed compositional and structural gradient along the thickness of the scale in Fig. 3 (although not presented for convenience, Cr_2_FeO_4_ spinel which can represent the observed outermost (Fe, Cr)-rich oxide is stabilized with pO_2_ over 10^− 23^). W0 and W5 exhibit very small difference in the constitution of oxides, whereas Laves-phase in W5 exhibits substantial stability against oxidation. Additionally, it is predicted to transform into μ-phase (Fe_7_W_6_) of higher W content with increasing pO_2_ due to the equilibrium with the matrix and other oxides, however not to transform into any W-based oxides within the present range of calculations.

The predicted stability of the second phases, whether they are Laves, μ-phase or others, can reflect the more numerous bright W-rich second phases concentrated at the interface of W5 from the early stage of oxidation as shown in Fig. 4b. And this could affect the advance of oxide toward the substrate, i.e. lead to slower oxidation kinetics as presented in Fig. 1. It could be also closely correlated to the formation of the characteristic particle of metallic W alloy within the scale in Fig. 6.

**Fig. 9:**
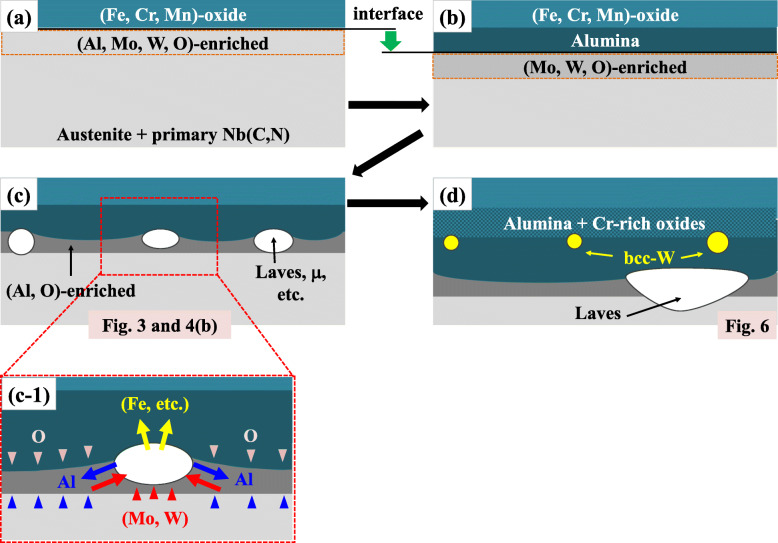
Schematic description on the progress of oxidation in W5: the oxidation proceeds in the sequence from (**a**) to (**d**) and (c-1) is the magnified area within the broken box in (**c**)

Fig. 9 schematically depicts the progress of oxidation in W5 which can be expected from the above discussions on thermodynamic equilibrium. In Fig. 9a, upon exposure to hot atmosphere, it would form (Fe, Cr, Mn)-rich oxides due to initially high pO_2_ at the bare metallic surface. And beneath this primary oxide layer, other metallic elements such as Al, Mo and W which have limited contribution to the formation of the primary scale would be enriched. Due to the protection by the primary scale, pO_2_ at this region should be much lower and alumina layer which is thermodynamically favored in lower pO_2_ should form as depicted in Fig. 9b. The intermediate Cr-richer layer of oxide between the two layers which was observed in Fig. 3 is omitted for brevity. Beneath the alumina layer, pO_2_ must be further lowered and the growth of scale is decelerated. Mo and W should be still enriched beneath the metal-oxide interface if the prior oxides should reject them due to insufficient miscibility. The process depicted in Fig. 9a-b should be common for all the alloys (W0-W5) and can be speculated from the phase equilibria in Fig. 8, although they only consider global equilibrium but not the local compositional variations of substrate (i.e. enrichment of some elements beneath the interface) in the progress of oxidation.

Figure 9c illustrates the oxidation stage corresponding to Figs. 3 and 4b. As shown by the alternating dark and bright volumes in the shallow region just beneath the interface, phase separation by precipitation of the bright W(and also Mo)-rich second phases takes place, which precedes that in the substrate because of the condensed Mo and W beneath the interface. Figure 9c-1 is the magnification of the region within the broken box in Fig. 9c and illustrates the elemental partitioning which is required for the phase separation. Mo and W should enter the second phases with their growth while other elements such as Al should be rejected from them. Thus the neighboring volumes should become rich in Al as confirmed in Table 2, which may partly contribute to their darkness in the STEI-DFs by compositional contrast. And they are also richer in O which infiltrated through the scale. These characteristic volumes should be transient structures which progressively transform to alumina with more O absorbed and could be temporarily termed as pro-alumina in this study. They were also found in W0 as shown in Fig. 4a, however the characteristic phase separation did not appear due to the less effectiveness of Mo in the formation of second phases as represented in Fig. 7a.

The existence and the characteristic compositions of pro-alumina could present some significant clues to the mechanisms of oxidation in AFAs. For instance, it can be known that the growth of alumina is governed by the reactions at the metal-oxide interface which should be controlled by the arrival of O passing through the priorly formed scale and by the elemental partitioning for the phase separation. In Table 2, the alumina layer exhibited very low levels of Mo and W, which implies that they should be rejected from pro-aluminas for their transform to alumina. An increasing fraction of W which is slower diffusant than Mo (Alberry and Haworth 1974) must retard the kinetics of this partitioning process at the interface and subsequently of the oxidation.

The fine metallic W alloy embedded in the scale (Fig. 6) indicates the instability of the W-rich second phases with increasing pO_2_ and the relative inertness of W against O in the given alloy system. The predicted phase equilibrium in Fig. 8b indeed shows the instability of Laves phase at increased pO_2_ and possible transformation into another phase, i.e. μ-phase. Additionally, at least in the given range of pO_2_ in Fig. 8, there was no W-based oxide predicted. Although it can fail in the exact identification of the initial and the final form of the W-rich second phase owing to various local and dynamic variability in the surface reaction as well as to incomplete databases, Fig. 8b is partly successful in the prediction of their transformation behavior with increased pO_2_. Through the transformation, an initial W-rich second phase would transform into a phase richer in W by rejection of Fe and other minor oxide-forming elements which react the surrounding oxygen, as indicated in Fig. 9c-1. With the growth of scale, the metal-oxide interface sweeps across the initial array of the second phases. Those second phases could be placed under increased pO_2_ as the more protective alumina layer passes by and the less protective oxide layers surrounds them. The continued rejection of Fe and other oxide-formers to feed the surrounding oxides can finally leave the fine precipitates of the metallic W alloy in Fig. 6, which is also depicted in Fig. 9d. Because of the phase equilibria predicted in Fig. 7a, the substrate region which was considerably apart from the initial metal-oxide interface should precipitate Laves-phases as confirmed in Fig. 6. And some of them would make contact with the advancing metal-oxide interface and also begin to interact with the growing oxides as depicted in Fig. 9d because of their incomplete stability under increased pO_2_.

## Conclusions

In this work, the effects of W on the oxidation of AFA were investigated with the alloys W0-W5 in which varying fractions of Mo were substituted with equivalent amounts of W. Weight gain after 336 h of exposure to hot atmosphere at 1053 K decreased as W content increased. From the microscopic observations on W0 and W5 which had the smallest and the largest amount of W respectively, the superior effectiveness of W on the retardation of scale growth was confirmed.

Increasing amount of W stabilized Laves-phase in matrix following the phase equilibria. W in the metallic substrate was condensed near the metal-oxide interface due to the loss of other elements by oxidation, which led to the profuse precipitation of W-rich second phases preferentially at the interface. By the pro-alumina which would transform to alumina and the W-rich second phases, the characteristic local feature, i.e. the alternating brightness beneath the interface was observed in the micrographs. Additionally, from the compositional variations by the phases near the interface, it could be interpreted that the required partitioning of W between the phases contributed to the slower advance of the interface. Thus, the initial fine dispersion of the W-rich second phases could act as obstacles against the interface.

In prolonged oxidation, fine particles of metallic W alloy were found within the scale of W5. They could be supposed to transform from the initial W-rich second phases at the metal-oxide interface with increasing oxygen partial pressure, which tells the instability of the W-rich second phases within the growing oxides.

## Data Availability

The data and the materials in the current manuscript cannot be shared because they also constitute a few on-going works, and the authors do not have the right to open them.
